# Controlling Autoreactivity of CD4 T Cells by Local Tolerance Induction

**DOI:** 10.1155/1998/83953

**Published:** 1998

**Authors:** Irmgard FöRster

**Affiliations:** lnstitute for Genetics University of Cologne Cologne Germany


*Developmental Immunology*, 1998, Vol. 6, pp. 89–94
Reprints available directly from the publisher
Photocopying permitted by license only

© 1998 OPA (Overseas Publishers Association)
N.V. Published by license under the
Harwood Academic Publishers imprint,
part of the Gordon and Breach Publishing Group
Printed in Malaysia

### *Minireview*

### Controlling Autoreactivity of CD4 T Cells by Local Tolerance Induction

IRMGARD FÖRSTER[a]*

[a]*Institute for Genetics, University of Cologne, Cologne, Germany*

*(Received 20 October 1996; In final form 30 May 1997)*

### INTRODUCTION

Autoimmune diseases are often caused by the inappropriate activation of CD4+ T cells specific for peripheral self-antigens. Since these cells recognize their target antigens in the context of MHC class II molecules on the surface of specialized antigen-presenting cells (APC), the induction of immunity, or alternatively tolerance, of CD4+ T cells depends on the release of antigens from peripheral tissues and uptake by APC. To study this process, transgenic mouse models have been established in which experimental self-antigens are expressed under the control of tissue-specific promoters. With the availability of T-cell-receptor (TCR)-transgenic mice specific for the respective antigen, the T-cell response toward such neo self-antigens can be followed directly during the development of the immune system (for review, see Hämmerling et al., 1993; Kruisbeek and Amsen, 1996; Mondino et al., 1996)

The transgenic mouse model described here has been originally established by D. Hanahan with the intention to study tissue-specific tumorigenesis following expression of a viral oncogene, the SV40 T antigen (Tag), under control of the rat insulin II gene promoter (RIP) (Hanahan, 1985). Independent lines of RIP-Tag transgenic mice were later shown to mount characteristic immune responses toward Tag, depending on the onset and level of Tag expression during ontogeny (Adams et al., 1987). Thus, RIP1-Tag2 (abbreviated RT2) mice with embryonic onset of Tag expression were found to establish systemic tolerance toward Tag, whereas other lines of mice with delayed onset of Tag expression developed a spontaneous autoimmune response against their pancreatic $\beta$ cells (Skowronski et al., 1990; Jolicoeur et al., 1994; Förster et al., 1995).

With the aim of generating Tag-specific TCR-transgenic mice to study the mechanism of induction of tolerance versus autoimmunity in this system, we identified and cloned a MHC class II restricted Tag-specific TCR that was expressed on Tag-specific CD4+ T cells isolated from pancreatic infiltrates of an autoimmune RIP1-Tag5 mouse (Förster et al., 1995).

*Corresponding author. Present address: Institut für Genetik, Weyertal 121, D-50931 Köln, Germany.

Genomic constructs encoding this Tag-specific TCR were injected into fertilized mouse oocytes and two independent lines of transgenic mice were obtained in which either a single copy of the TCR $\alpha$-chain gene and two copies of the TCR $\beta$-chain gene (TCR1 mice) or multiple copies of both TCR $\alpha$ and $\beta$ (TCR2 mice) were cointegrated into the genome. With the help of an anti-idiotypic antibody specific for the transgenic TCR, it could be demonstrated that TCR1 mice expressed the transgenic receptor on no more than 0.5% of thymocytes and 10% of peripheral $CD4^+$ T cells, whereas the majority of peripheral T cells expressed endogenous TCR. In contrast, TCR2 mice carried the transgenic TCR on almost all thymocytes and 90% of peripheral T cells (Förster et al., 1995). The reason for this differential expression of the transgenic TCR in TCR1 versus TCR2 mice is presently unknown but most likely depends on position effects of the transgene integration site.

### ESTABLISHMENT OF PERIPHERAL TOLERANCE DEPENDS ON THE FREQUENCY OF AUTOREACTIVE T CELLS

When the two different Tag-specific TCR-transgenic lines were crossed to the tolerant RT2 line, we found that only RT2/TCR1 mice established tolerance to Tag, as evident by deletion of most of the transgenic T cells and functional inactivation of the remaining ones. In contrast, RT2/TCR2 double-transgenic mice failed to develop peripheral T-cell tolerance. This result could be attributed to the different frequencies of autoreactive T cells in TCR1 versus TCR2 mice rather than intrinsic differences between the two lines, as demonstrated by two independent experimental approaches. In the first series of experiments, the frequency of Tag-specific T cells in TCR2 mice was reduced by generation of mixed bone-marrow chimaeras in which bone marrow derived from TCR2 mice was mixed at various ratios with bone marrow from nontransgenic mice and transferred into sublethally irradiated RT2 or negative control mice.

Analyzing the tolerance status of these chimaeras, we could demonstrate that transgenic ($Id^+$) T cells derived from TCR2 mice were susceptible to tolerance induction when present at low frequencies (i.e., less than 15% of peripheral $CD4^+$ T cells) (Förster et al., 1995). Conversely, in a different set of experiments, the frequency of $Id^+$ T cells in TCR1 mice was increased to 100% by breeding of the TCR1 line into the RAG-1-deficient background (Mombaerts et al., 1992). Interestingly, even in the absence of endogenous TCR rearrangements, the absolute number of transgenic T cells in TCR1/RAG-$1^{-/-}$ mice remained low, that is, the total number of thymocytes in these mice was only 2-3 $\times$ $10^6$ cells, and the number of peripheral $Id^+$ T cells in TCR1/RAG-$1^{-/-}$ mice was no more than a 4-5-fold increase over that in normal TCR1 mice. Even though the increase in the absolute number of Tag-specific T cells in TCR1/RAG-$1^{-/-}$ mice was moderate, RT2/TCR1 double-transgenic mice in the RAG-$1^{-/-}$ background developed an autoimmune response that was more severe than the one originally observed in RT2/TCR2 double-transgenic mice (Förster and Lieberam, 1996).

As shown in Figures 1(c) to 1(f), transgenic $CD4^+$ T cells as well as a few $CD8^+$ T cells expressing the same MHC class II restricted TCR infiltrated into the pancreatic islets and destroyed most of the Tag-expressing $\beta$ cells despite constitutive expression of the viral oncogene, which in tolerant mice invariably causes the formation of $\beta$-cell tumors by the age of 10-12 weeks; for comparison, see Figures 1(a) and 1(b), depicting part of a tumorigenic islet in a tolerant RT2/TCR1 mouse. Remarkably, the autoimmune response in RT2/TCR1/RAG-$1^{-/-}$ mice strongly reduced or even prevented Tag-dependent tumor formation, and in about one-third of the mice led to the development of diabetes with blood glucose levels higher than 300 mg/dl (Figure 2). Mice that did not become diabetic remained normoglycemic during the observation period (7-14 weeks of age), whereas tolerant RT2/TCR1 mice older than 10 weeks inevitably developed hypoglycemia due to the formation of Tag-induced insulinomas (Figure 2).

The failure of tolerance induction in mice with a high frequency of autoreactive CD4 T cells may be explained by a limiting amount of the tolerizing antigen expressed in peripheral tissues and the

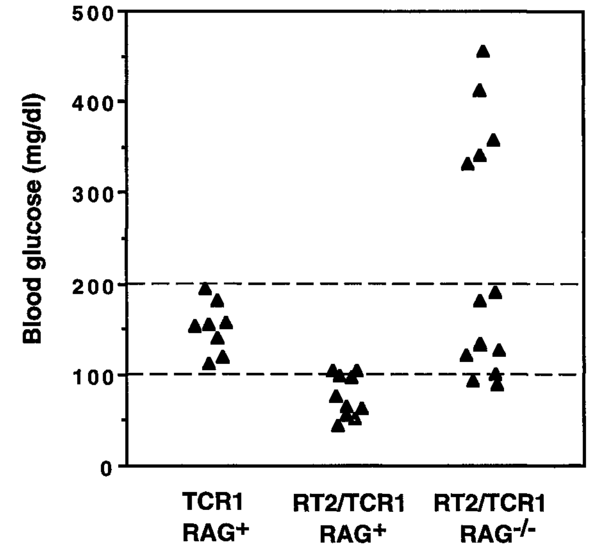

FIGURE 2 Development of diabetes in RT2/TCR1/RAG-$1^{-/-}$ mice. Shown are the blood glucose levels of groups of 7 TCR1/RAG-$1^{+}$, 10 RT2/TCR1/RAG-$1^{+}$, and 14 RT2/TCR1/RAG-$1^{-/-}$ mice. All mice were between 7 and 14 weeks of age. Development of diabetes was observed both in young and older RT2/TCR1/RAG-$1^{-/-}$ mice.

### ONTOGENETIC TIMING OF TOLERANCE INDUCTION

Considering the substantial autoreactive potential of the transgenic Tag-specific T cells, tolerant RT2/TCR1 double-transgenic mice offer a unique possibility to unravel the naturally occurring mechanism of peripheral $CD4^+$ T-cell tolerance induction. As mentioned before, the initial analysis of adult RT2/TCR1 mice demonstrated that the majority of $Id^+$ T cells in these mice were deleted and the remaining Tag-specific T cells appeared functionally impaired. By examining tolerance induction in adolescent mice, we noticed that deletion of the autoreactive T cells was not apparent before the age of 3 weeks (Förster and Lieberam, 1996). Rather, $Id^+$ T cells derived from mesenteric lymph nodes (LN) of 3-week-old RT2/TCR1 mice were found to be normally responsive to the Tag peptide (362-384) that is specifically recognized by the transgenic TCR. Since Tag is expressed in these mice starting from embryonic day 10, it was important to assess whether the nontolerant Tag-specific T cells in postnatal mice were ignorant of the pancreatic Tag or were actively undergoing an immune response. Indeed, upregulation of the early activation marker CD69 (Testi et al., 1989) as well as the memory T-cell marker CD44 (DeGrendele et al., 1996) on $Id^+$ T cells of RT2/TCR1 mice could be noticed as early as 1 week after birth.

Consistent with activation, the Tag-specific T cells also increased in size and downregulated the L-selectin LN homing receptor. Activation was maximal between 2 and 3 weeks of age, at which point approximately two-thirds of the $Id^+$ T cells expressed the CD69 activation marker. Starting from 3 weeks of age until adulthood, 80-90% of the transgenic T cells were deleted from the peripheral lymphocyte pool, whereas half of the remaining cells continued to exhibit an activated phenotype. Analyses of cell turnover by *in vivo* labeling with the thymidine analogue bromodeoxyuridine were consistent with the interpretation that most of the activated $Id^+$ T cells in adult mice represent relatively recent thymic emigrants with an average lifespan of 1 to 2 weeks (Förster and Lieberam, 1996, and unpublished data).

### ARE DRAINING LYMPH NODES THE SITES OF TOLERANCE INDUCTION?

The presence of activated $CD69^+$, Tag-specific T cells in mesenteric LN suggests that these cells may have been stimulated by APC presenting the appropriate Tag peptide in the LN themselves. Since expression of the Tag transgene has not been detected in LN (Jolicoeur et al., 1994), an alternative explanation could be that APC internalize Tag protein in the pancreas and migrate to the local draining LN. In the human, the mesenteric LN as well as several other peritoneal LN have been demonstrated to be within the lymphoid drainage pathway of the pancreas (Evans and Ochsner, 1954). Therefore, a panel of different LN from various locations was examined for the presence of activated Tag-specific T cells in the early phase of tolerance induction. Interestingly, $CD69^+Id^+$ T cells could be detected only in LN located in the peritoneal cavity (mesenteric, infrapancreatic, and paraaortic LN) and to a lower extent in Peyer's patches of the small intestine but not in more remote locations like axillary, cervical, and inguinal LN. Nevertheless, Tag-specific T cells isolated from LN outside the peritoneal cavity expressed slightly elevated levels of CD44 and were found to be as unresponsive to Tag stimulation *in vitro* as mesenteric LN cells (Förster and Lieberam, 1996).

Since transient insulitis was observed in young RT2/TCR1 mice prior to tolerance induction, it cannot be excluded that the Tag-specific T cells had been activated initially in the pancreas before they reached the draining LN. However, the current knowledge on the migration properties of lymphocytes (Smith and Ford, 1983; Mackay et al., 1990) rather favors the hypothesis that naive Tag-specific T cells first recognize their target antigen on APC located in the draining LN of the pancreas before they are enabled to enter the pancreatic tissue as activated T cells. Following tolerance induction, some of the transgenic T cells may then recirculate throughout the immune system as CD69-negative, anergic cells with slightly elevated expression of CD44.

### CONCLUDING REMARKS

A key question concerning the mechanism of tolerance induction of $CD4^+$ T cells is how and where the autoantigen is presented to the T cells. The model proposed suggests that the antigen may be delivered to the draining LN through uptake by local APC. This pathway requires that some of the antigen must be released from peripheral tissues, perhaps through naturally occurring cell death (apoptosis) or by an unknown mechanism of secretion of otherwise intracellular proteins. In the case of Tag, it cannot be excluded that the oncogenic activity of the protein may aid in inducing cellular abnormalities in the pancreatic $\beta$ cells that promote uptake of Tag by macrophages or dendritic cells. Lack of antigen presentation in draining LN may prevent the induction of systemic tolerance and rather result in immunologic ignorance, that is, failure to recognize secluded antigens (Ohashi, 1994). As demonstrated previously, activation of potentially autoreactive T cells specific for such secluded antigens may cause severe autoimmune responses (Ohashi et al., 1991; Oldstone et al., 1991). Therefore, understanding the mechanism of peripheral tolerance induction via appropriate delivery of autoantigens to local LN may be of major importance for the prevention of autoimmune diseases.

### Acknowledgments

This work was supported by the Deutsche Forschungsgemeinschaft through SFB243, the Land Nordrhein Westfalen through the Bennigsen-Foerder Preis, and the Human Frontiers of Sciences Program.

### *References*

Adams T.E., Alpert S., and Hanahan D. (1987). Non-tolerance and autoantibodies to a transgenic self antigen expressed in pancreatic beta cells. Nature **325**: 223-228.

DeGrendele H.C., Estess P., Picker L.J., and Siegelman M.H. (1996). CD44 and its ligand hyaluronate mediate rolling under physiologic flow: A novel lymphocyte-endothelial cell primary adhesion pathway. J. Exp. Med. **183**: 1119-1130.

Evans B.P., and Ochsner A. (1954). The gross anatomy of the lymphatics of the human pancreas. Surgery **36**: 177-191.

Förster I., Hirose R., Arbeit J.M., Clausen B.E., and Hanahan D. (1995). Limited capacity for tolerization of CD4+ T cells specific for a pancreatic beta cell neo-antigen. Immunity **2**: 573-585.

Förster I., and Lieberam I. (1996). Peripheral tolerance of CD4 T cells following local activation in adolescent mice. Eur. J. Immunol. **26**: 3194-3202.

Hämmerling G.J., Schönrich G., Ferber I., and Arnold B. (1993). Peripheral tolerance as a multi-step mechanism. Immunol. Rev. **133**: 93-104.

Hanahan D. (1985). Heritable formation of pancreatic beta-cell tumours in transgenic mice expressing recombinant insulin/simian virus 40 oncogenes. Nature **315**: 115-122.

Jolicoeur C., Hanahan D., and Smith K.M. (1994). T cell tolerance towards a transgenic $\beta$ cell antigen and transcription of endogenous pancreatic genes in thymus. Proc. Natl. Acad. Sci. USA **91**: 6707-6711.

Kruisbeek A.M., and Amsen D. (1996). Mechanisms underlying T-cell tolerance. Curr. Opin. Immunol. **8**: 233-244.

Lafaille J.J., Nagashima K., Katsuki M., and Tonegawa S. (1994). High incidence of spontaneous autoimmune encephalomyelitis in immunodeficient anti-myelin basic protein T cell receptor transgenic mice. Cell **78**: 399-408.

Mackay C.R., Marston W.L., and Dudler L. (1990). Naive and memory T cells show distinct pathways of lymphocyte recirculation. J. Exp. Med. **171**: 801-817.

Mombaerts P., Iacomini J., Johnson R.S., Herrup K., Tonegawa S., and Papaioannou V.E. (1992). RAG-1-deficient mice have no mature B and T lymphocytes. Cell **68**: 869-877.

Mondino A., Khoruts A., and Jenkins M.K. (1996). The anatomy of T-cell activation and tolerance. Proc. Natl. Acad. Sci. USA **93**: 2245-2252.

Ohashi P.S. (1994). Ignorance is bliss. Immunologist **2**: 87-92.

Ohashi P.S., Oehen S., Buerki K., Pircher H., Ohashi C.T., Odermatt B., Malissen B., Zinkernagel R.M., and Hengartner H. (1991). Ablation of "tolerance" and induction of diabetes by virus infection in viral antigen transgenic mice. Cell **65**: 305-317.

Oldstone M.B., Nerenberg M., Southern P., Price J., and Lewicki H. (1991). Virus infection triggers insulin-dependent diabetes mellitus in a transgenic model: Role of anti-self (virus) immune response. Cell **65**: 319-31.

Skowronski J., Jolicoeur C., Alpert S., and Hanahan D. (1990). Determinants of the B-cell response against a transgenic autoantigen. Proc. Natl. Acad. Sci. USA **87**: 7487-7491.

Smith M.E., and Ford W.L. (1983). The recirculating lymphocyte pool of the rat: A systematic description of the migratory behaviour of recirculating lymphocytes. Immunology **49**: 83-94.

Testi R., Phillips J.H., and Lanier L.L. (1989). Leu 23 induction as an early marker of functional CD3/T cell antigen receptor triggering. Requirement for receptor cross-linking, prolonged elevation of intracellular [Ca++] and stimulation of protein kinase C. J. Immunol. **142**: 1854-1860.

